# Association of a new *FCN3* haplotype with high ficolin-3 levels in leprosy

**DOI:** 10.1371/journal.pntd.0005409

**Published:** 2017-02-27

**Authors:** Fabiana Antunes Andrade, Marcia Holsbach Beltrame, Valéria Bumiller Bini, Letícia Boslooper Gonçalves, Angelica Beate Winter Boldt, Iara Jose de Messias-Reason

**Affiliations:** 1 Laboratory of Molecular Immunopathology, Department of Clinical Pathology, HC/UFPR, Curitiba, PR, Brazil; 2 Laboratory of Human Molecular Genetics, Department of Genetics, UFPR, Curitiba, PR, Brazil; Fondation Raoul Follereau, FRANCE

## Abstract

Leprosy is a chronic inflammatory disease caused by *Mycobacterium leprae* that mainly affects the skin and peripheral nervous system, leading to a high disability rate and social stigma. Previous studies have shown a contribution of genes encoding products of the lectin pathway of complement in the modulation of the susceptibility to leprosy; however, the ficolin-3/*FCN3* gene impact on leprosy is currently unknown. The aim of the present study was to investigate if *FCN3* polymorphisms (rs532781899: *g*.*1637delC*, rs28362807: *g*.*3524_3532insTATTTGGCC* and rs4494157: *g*.*4473C>A*) and ficolin-3 serum levels play a role in the susceptibility to leprosy. We genotyped up to 190 leprosy patients (being 114 (60%) lepromatous), and up to 245 controls with sequence-specific PCR. We also measured protein levels using ELISA in 61 leprosy and 73 controls. *FCN3* polymorphisms were not associated with disease, but ficolin-3 levels were higher in patients with FCN3 *2B1 (C*ins*A) haplotype (p = 0.032). Median concentration of ficolin-3 was higher in leprosy *per se* (26034 ng/mL, p = 0.005) and lepromatous patients (28295 ng/mL, p = 0.016) than controls (18231 ng/mL). In addition, high ficolin-3 levels (>33362 ng/mL) were more common in leprosy *per se* (34.4%) and in lepromatous patients (35.5%) than controls (19.2%; p = 0.045 and p = 0.047, respectively). Our results lead us to suggest that polymorphisms in the *FCN3* gene cooperate to increase ficolin-3 concentration and that it might contribute to leprosy susceptibility by favoring *M*. *leprae* infection.

## Author summary

Leprosy is considered a neglected disease and still a public health problem in many countries where it was not yet eliminated, leading to a high disability rate and social stigma. The molecular mechanisms of *M*. *leprae* infection and immune evasion are still poorly known, raising the need for studies that may contribute to a better understanding of leprosy etiology, as well as improvement in diagnosis and treatment. Ficolin-3 is a soluble molecule of the innate immune system that recognizes a wide range of pathogen-associated molecular patterns leading to complement activation and phagocytosis. We observed high concentration of ficolin-3 in leprosy patients, likely caused by polymorphisms present in intronic regions of *FCN3* gene, which may contribute to leprosy susceptibility by favoring *M*. *leprae* infection. This is the first study addressing *FCN3* polymorphisms and ficolin-3 levels in leprosy, indicating it as a good candidate biomarker associated with the host response against *M*. *leprae*.

## Introduction

Leprosy is a chronic infectious disease caused by *Mycobacterium leprae* that mainly affects the skin and peripheral nerves [1] and can cause progressive and permanent damage, if untreated. Despite the disease elimination in 119 of the 122 countries where it was considered a public health problem, Brazil still ranked second in the world, behind India and accounts for 92% of leprosy cases in the Americas [2].

Upon exposure to *M*. *leprae*, most individuals are intrinsically resistant to infection. Among those who are susceptible, infection may progresses to a wide spectrum of manifestations, with two polar forms: the tuberculoid leprosy and the lepromatous leprosy. Tuberculoid leprosy is characterized by strong cell-mediated immunity, type 1 cytokine profile, low bacillary load and localized lesions. On the other hand, lepromatous leprosy is characterized by low cellular response, type 2 cytokine profile, high bacillary load and disseminated lesions [3]. There is enough evidence to suggest that susceptibility to leprosy and to different clinical manifestations is markedly influenced by host genetic factors [3–6].

Ficolins (Ficolin-1 or M-Ficolin, Ficolin-2 or L-Ficolin and Ficolin-3 or H-ficolin) are soluble molecules of the innate immune system that recognize a wide range of pathogen-associated molecular patterns (PAMPs) [7,8]. Ficolins form complexes with MASPs (MBL-associated serine proteases or MASPs) and activate complement through the lectin pathway, leading to opsonization and phagocytosis of pathogens, and stimulating the production of inflammatory cytokines and nitric oxide [9]. Most active ficolins are composed of four trimeric subunits. Each monomer is formed by an N-terminal region, a collagen-like domain and a fibrinogen-like domain; important in the oligomerization process, in the MASP/phagocyte interaction and in the recognition of specific PAMPs in pathogens, respectively [9].

Ficolins 1, 2 and 3 are encoded by *FCN1* and *FCN2* genes on 9q34 and *FCN3* on 1p36.11, respectively [10]. In previous studies, we demonstrated that *FCN2* gene haplotypes associated with normal ficolin-2 levels have a protective effect against leprosy [11] and that *FCN1* gene *-271DelT*, *-399A*, *-542G*, *-1981A* polymorphisms were associated with susceptibility to leprosy [12]. *FCN3* comprises eight exons, one of them being an alternative exon (exon 4). Both *FCN3* transcripts, with and without exon 4, occur especially in the lung, but also in the liver, heart, kidney, adrenal gland, breast, spleen, thyroid and visceral adipose tissue, but the shorter transcript is less abundant [13]. Exons are highly conserved: although 164 polymorphic noncoding variants are currently listed in Ensembl, all coding DNA variations (including those synonymous) occur at global frequencies below 1%. The *g*.*1637delC* variant (rs532781899) in exon 5 is actually the only one reported to be polymorphic at the global scale [14]. It causes a frameshift, leading to premature termination of the translation product. This generates a truncated protein, unable to perform PAMP recognition and complement activation, which may be associated with repetitive infections in some individuals [15–18].

Ficolin-3 has 299 amino acids and is the most abundant ficolin in serum, with a median concentration of ~19500 ng/mL (range 3000–60300 ng/mL) [17,19]. Low ficolin-3 concentration in serum has already been associated with the pathophysiology of sarcoidosis [20], chemotherapy-related infections in children [21], Crohn's disease [22] and heart failure [23]. On the other hand, high ficolin-3 levels were associated with Systemic Lupus Erythematosus [24], ovarian tumors [25] and seem to be a risk factor for shorter graft survival in kidney transplantation [26]. The impact of *FCN3* polymorphisms in other diseases has yet to be explored.

In this work, we investigated whether *FCN3* polymorphisms and ficolin-3 serum levels play a role in the susceptibility to leprosy and observed an association between high ficolin-3 levels in serum and the disease.

## Material and methods

### Ethics statement

The study was approved by Human Research Ethics Committee, Health Sciences Sector at Federal University of Parana, Brazil (approval number: 218.104).

### Subjects and samples

Study subjects comprised consecutive outpatients from the Hospital de Clínicas, Federal University of Paraná, State Health Department of Paraná, and inpatients from the Sanitary and Dermatologic Hospital of Paraná, both located in Curitiba, southern Brazil. For all 190 patients (38.4% female; 82.3% Euro-Brazilian, 17.7% Afro-Brazilian; average age of 51.5 years, range 18–94), leprosy was diagnosed on the basis of the clinical and histopathological features of affected lesions and classified according to the criteria of Ridley and Jopling [27]. The initial diagnosis was lepromatous leprosy for 114 (60%), tuberculoid leprosy for 15 (7.9%), and borderline leprosy for 28 patients (14.7%); 10 patients (5.3%) had an undetermined form of leprosy and 23 (12.1%) were unspecified. As control subjects, 245, unrelated, symptom-free blood donors from HEMEPAR (Centro de Hematologia e Hemoterapia do Paraná) were assessed (53% female; 80% Euro-Brazilian, 15% Afro-Brazilian; average age of 37.7 years, range 18–61). Patients and control subjects had a similar socioeconomic status, were from the same geographical area, and shared the same ethnic background. All patients and control subjects provided written informed consent.

### *FCN3* genotyping

DNA extraction was performed using QIAamp DNA extraction kits (Qiagen) according to the manufacturer’s instructions. Three *FCN3* SNPs were assessed by sequence-specific amplification method (PCR-SSP), being: *g*.*1637delC* (rs532781899) in exon 5; *g*.*3524_3532insTATTTGGCC* (rs28362807) in intron 5 and *g*.*4473C>A* (rs4494157) in intron 7 (Table 1). Although there are other noncoding polymorphisms not in LD with those selected, they do not tag a haplotype block in the Iberian population (data from the 1000 Genomes project), which is representative for most Euro-Brazilians, as do the intronic polymorphisms chosen for this study. FCN3_Ex5_1637del_R or FCN3_Ex5_1637C_R were conjugated with FCN3_Ex5_F primer to generate a fragment of 748 bp. An amplification control fragment of 500 bp of *FCN2* gene was simultaneously generated. FCN3_In5_3524_3532del_F or FCN3_In5_3524_3532ins_F were conjugated with FCN3_In7_4473A_R or FCN3_In7_+4473C_R primer to generate a fragment of 984 bp. A control fragment of 431 bp of *HGH* gene was simultaneously generated. The intron 5_intron 7 haplotypes were determined without having to infer their phase on the chromosomes due to the PCR-SSP approach with primers annealing on two different SNPs (S1 Fig).

**Table 1:** Primers used for *FCN3* sequence-specific amplification.

dbSNP	Gene region	Alleles^a^	5’-3’ forward primer		5’-3’ reverse primer		Amplicon
rs532781899	Exon 5	*g*.*1637C*	FCN3_Ex5_F	TAGGGTGGGATCTCTGCTTG	FCN3_Ex5_1637C_R	TGTCACAAAAGACTGGGAGGG	748 bp^b^
		*g*.*1637del*			FCN3_Ex5_1637del_R	TGTCACAAAAGACTGGGAGGC	
rs28362807	Intron 5	*g*.*3524_3532del*	FCN3_In5_3524_3532del_F	GCCACCAAGCGTTCTTGG			984 bp^c^
		*g*.*3524_3532ins*	FCN3_In5_3524_3532ins_F	CCACCAAGCGTGGCCAAA			
rs4494157	Intron 7	*g*.*4473C*			FCN3_In7_4473C_R	GAGGAGGAAACTGAGGCTCAG	
		*g*.*4473A*			FCN3_In7_4473A_R	GAGGAGGAAACTGAGGCTCAT	

^a^Position of polymorphisms are counted with respect to the *FCN3* translation start site with the A of ATG being +1

^b^PCR-SSP endogenous control: 500 bp of the *FCN2* gene (forward primer 5’GCCAGGCCTCAGGTATAAAG3’ and reverse primer 5’AAAGGGTTGATTGCGGAAAC3’)

^c^PCR-multiplex endogenous control: 431 bp of the *HGH* gene (forward primer 5’TGCCTTCCCAACCATTCCCTTA3’ and reverse primer 5’CCACTCACGGATTCTGTTGTGTTTC3’).

PCR was carried out in a final volume of 15 μl in a T100^TM^ thermocycler (BioRad). PCR conditions were as follows: 0.7 μM for exon 5 and 0.2 μM for intron 5 and 7 SSP primers and 0.1 μM control primers, 1 × Coral Load PCR buffer (Qiagen, Hilden, Germany), 2.0–1.75 mM MgCl_2_ (only for exon 5 primers reaction, Qiagen, Hilden, Germany), 1.5% glycerol, 0.2 mM deoxyribonucleotide triphosphate (dNTP) (Invitrogen, São Paulo, Brazil), 0.5% Q Solution (only for intron 5 and 7 primers reaction, Qiagen, Hilden, Germany), 0.03 U/μl of Taq polymerase (Invitrogen, São Paulo, Brazil), 20 ng/μl DNA and water to complete the final volume. The amplification protocol starts with a 3 min denaturation step at 96°C, followed by 35 cycles of 15 sec at 94°C, 30 sec at the specific annealing temperature and 30 sec at 72°C, concluding with 5 min at 72°C in the final DNA extension step. Annealing temperature decreased every 10 cycles (64°C, 62°C and 60°C; 60°C, 58°C and 56°C for exon 5 and intron5_7 primers reaction, respectively), according to a previously published “touch-down” strategy which assures higher specificity to the amplification, while providing a larger amount of the desired PCR product [28]. The haplotypes defined by two SNPs, amplified by a pair of SSPs, were identified by the presence or absence of specific bands after agarose gel electrophoresis. Control bands informed on the quality of the reactions.

### Ficolin-3 concentration assay

We measured ficolin-3 concentrations in 1:250 diluted sera (1:150 or 1:50 when necessary) of 61 patients and 73 controls with the same proportion of selected *FCN3* genotypes, using the enzyme-linked immune sorbent assay HK 340 (Hycult Biotechnology, Uden, The Netherlands). Relative low ficolin-3 concentration was defined as <10368 ng/mL, which corresponded to the 20th percentile, and high levels as >33362 ng/mL, corresponding to the 80th percentile among controls.

### Statistical analyses

Genotype and allele frequencies were obtained by direct counting. The hypothesis of Hardy–Weinberg equilibrium was verified using the approach of Guo and Thompson implemented in the ARLEQUIN software package version 3.1 (http://anthro.unige.ch/arlequin/). Tests of independence between patients and controls, as well as between patients with the lepromatous and non-lepromatous forms (tuberculoid, borderline and undetermined form of leprosy), were performed using Fisher exact test. Ficolin-3 levels were compared between the groups using nonparametric Mann-Whitney/Kruskal–Wallis tests using GraphPad Prism 3.0 software package. Two-tailed P-values less than 5% were considered significant. Logistic regression models were used to adjust results for age, sex and ethnic group distribution, using STATA v.9.2 (Statacorp, USA). Due to the sample size, statistical analyzes were performed between lepromatous leprosy and non-lepromatous patients (which included tuberculoid, borderline and undetermined leprosy). Clinical forms of leprosy was compared with healthy controls since this approach could reveal subtle differences not apparent when comparing them just with leprosy *per se*.

## Results

### *FCN3* polymorphisms and haplotypes

*FCN3* genotype distribution was in Hardy-Weinberg equilibrium. The allelic frequencies in Euro-Brazilian and Afro-Brazilian patients and controls did not differ from those reported in the HapMap project for CEU (North-Americans of Northern and Western European ancestry from Utah) and YRI (Yoruba in Ibadan, Nigeria) populations [29]. There was no difference in the allelic and genotypic frequencies between controls and leprosy patients, as well as lepromatous and non lepromatous groups (Table 2). Importantly, due to the very low frequencies of *g*.*1637del*, our study was underpowered in detecting associations with this SNP. Euro-Brazilians and Afro-Brazilians as well as males and females also had similar allelic and genotypic frequencies.

**Table 2:** *FCN3* genotype, allele and haplotype frequencies (%).

dbSNP		Controls	Leprosy *per se*	Lepromatous	Non-lepromatous
**rs532781899**		**N = 245**	**N = 190**	**N = 144**	**N = 53**
Genotype	*g*.*1637C/1637C*	96.0	95.0	95.0	94.0
	*g*.*1637del/1637C*	4.0	5.0	5.0	6.0
		**n = 490**	**n = 380**	**n = 228**	**n = 106**
Allele	*g*.*1637C*	97.8	97.6	97.4	97.2
	*g*.*1637del*	2.2	2.4	2.6	2.8
**rs28362807**		**N = 146**	**N = 149**	**N = 87**	**N = 42**
Genotype	*g*.*3524_3532del/3524_3532del*	51.0	52.0	49.0	55.0
	*g*.*3524_3532del/3524_3532ins*	42.0	42.0	43.0	43.0
	*g*.*3524_3532ins/3524_3532ins*	7.0	6.0	8.0	2.0
		**n = 292**	**n = 298**	**n = 174**	**n = 84**
Allele	*g*.*3524_3532del*	72.3	72.8	71	76.2
	*g*.*3524_3532ins*	27.7	27.2	29	23.8
**rs4494157**		**N = 146**	**N = 149**	**N = 87**	**N = 42**
Genotype	*g*.*4473C/4473C*	55.0	58.0	53.0	64.0
	*g*.*4473C/4473A*	40.0	38.0	40.0	36.0
	*g*.*4473A/4473A*	4.0	5.0	7.0	-
		**n = 292**	**n = 298**	**n = 174**	**n = 84**
Allele	*g*.*4473C*	75.7	76.5	73	82.1
	*g*.*4473A*	24.3	23.5	27	17.9
**Phylogenetic nomenclature**	**Haplotype**	**n = 292**	**n = 298**	**n = 174**	**n = 84**
*2ª	C del C	71.6	71.1	69	73.8
*2B1	C ins A	24.3	23.5	27	17.9
*1	C ins C	2.74	3.7	2.3	5.9
*2B2.*2A*	del del C	0.68	1.7	1.7	2.4
*2B2	del ins C	0.68	-	-	-

N: number of individuals. n: number of chromosomes. dbSNP: nomenclature according to the Single Nucleotide Polymorphism database. Unspecified leprosy patients were excluding when clinical forms of the disease were compared.

**Fig 1:**
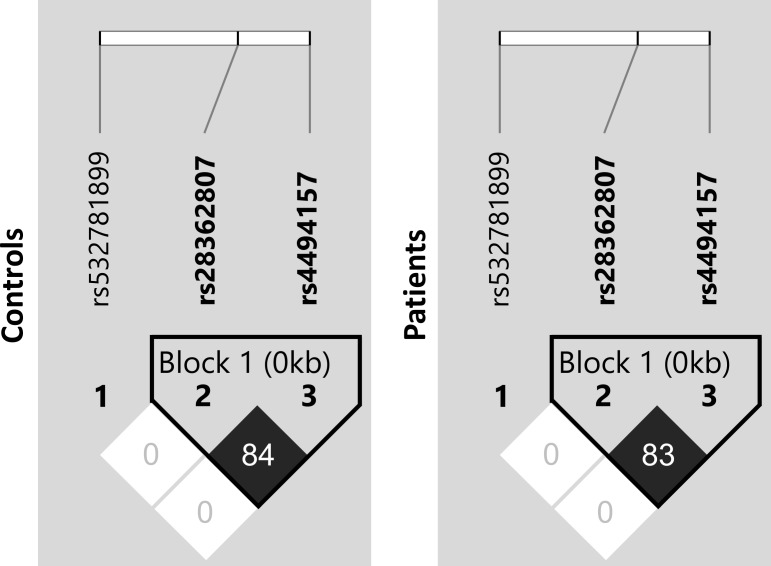
Linkage disequilibrium between *FCN3* single nucleotide polymorphisms. LD was calculated based on the data for controls and leprosy patients. Black squares represent high LD and white low LD as measured by the correlation coefficient (r^2^) between sites, which values are shown inside of the squares. SNP identifiers are indicated on the abscissas.

**Fig 2:**
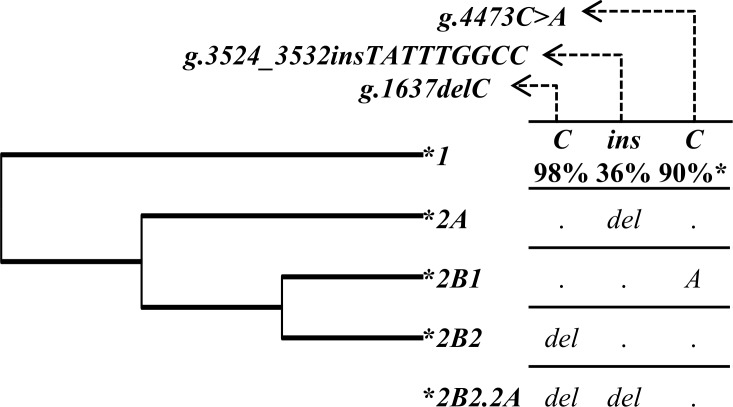
Phylogenetic tree of *FCN3* haplotypes. The maximum parsimony tree was rooted on the haplotype shared with *Pan troglodytes* (ENSPTRT00000000796), named as *1, and the derived haplotype as *2, following the schema numerals/letters/ numerals, if they diverge further [30]. Recombinants are named according to the most common inferred parental haplotypes, separated by a dot [31]. SNPs in haplotypes were ordered according to their chromosomal position. *Allelic frequency in the African population, from the 1000 Genomes project [14].

There was strong linkage disequilibrium (LD) between the two non-coding SNPs rs28362807 (*g*.*3524_3532insTATTTGGCC*, intron 5) and rs4494157 (*g*.*4473C>A*, intron 7), as indicated by the correlation coefficient values (r^2^) (Fig 1). The low r^2^ values observed for *g*.*1637delC* SNP reflect the completely discrepant frequencies of this SNP (an uncommon deletion) in comparison to the other two SNPs. Different combinations of investigated polymorphisms (*g*.*1637delC*, *g*.*3524_3532insTATTTGGCC* and *g*.*4473C>A*) resulted in 5 observed haplotypes, one of them possibly recombinant. According to the degree of sequence identity with the *Pan troglodytes FCN3* gene sequence, the most probable ancestral haplotype (named as *1) is formed by *g*.*1637C*, *g*.*3524_3532ins* and *g*.*4473C* alleles (for short, C*ins*C*)* (Fig 2). The *2A (C*del*C) haplotype was the most frequent, with 69–74% frequency in all investigated groups followed by *2B1 (C*ins*A) haplotype (18–27%). Other haplotypes, including *2B2 (*delins*C), harboring the deletion in exon 5, were rather uncommon (Table 2). All the alleles found in our Afro-Brazilian sample were also found in all African population from NCBI and 1000 Genomes Project [14,29], giving us clues about the likely African origin of these polymorphisms.

### Association of *FCN3* polymorphisms and ficolin-3 levels

**Fig 3:**
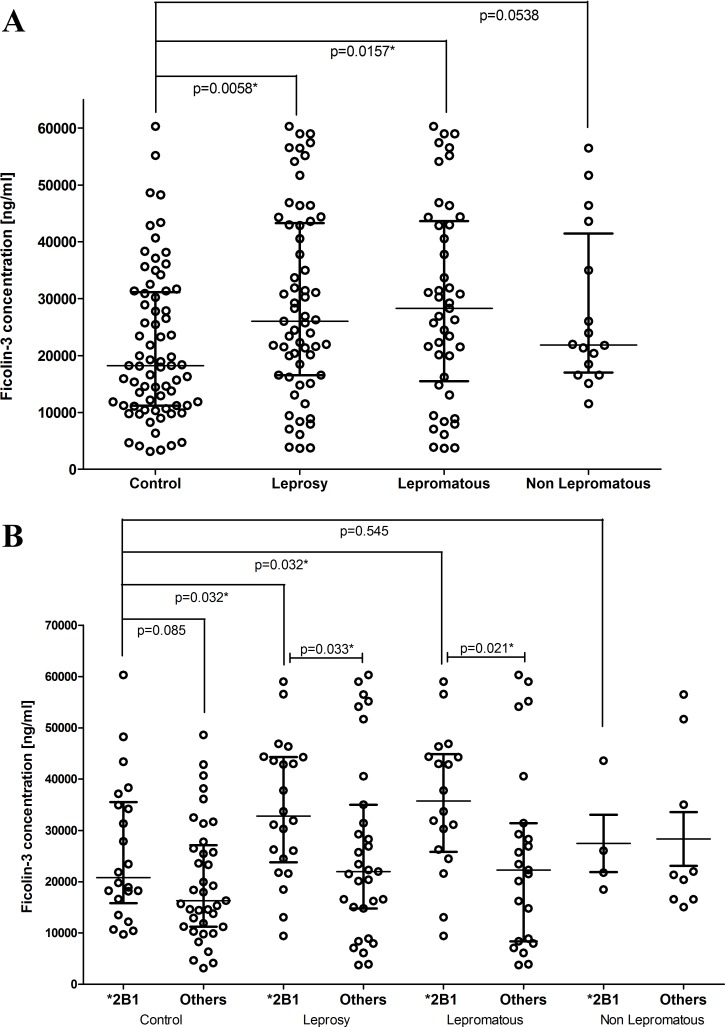
Distribution of Ficolin-3 levels in the investigated groups. Ficolin-3 levels in (a) the investigated groups and (b) in individuals with and without the *FCN3* *2B1 haplotype. Comparisons were made using Mann-Whitney test. * indicates significant p values (<0.05). Bars indicate median and interquartile values.

The median level of ficolin-3 observed in the control group (18231 ng/mL [3129–60300 ng/mL]) is in good agreement with published data in adults (~19500 ng/mL; [19]). The concentration of ficolin-3 in serum was higher in leprosy per se (26034 ng/mL, p = 0.005, OR 6.80 [1.65–28]) and lepromatous patients (28295 ng/mL, p = 0.016, OR 6.77 [1.43–32]) compared with controls (18231 ng/mL), even after correction for age, sex and ethnic group, but did not differ between lepromatous and non lepromatous groups (Fig 3A). In addition, high ficolin-3 levels (>33362 ng/mL) were more common in leprosy *per se* (34.4%) and in lepromatous patients (35.5%) than controls (19.2%; p = 0.045 and p = 0.047, respectively), while the frequency of low ficolin-3 concentrations (<10368 ng/mL) did not differ between the groups (Table 3). To assess whether the polymorphisms *g*.*1637delC* (rs532781899); *g*.*3524_3532insTATTTGGCC* (rs28362807) and *g*.*4473C>A* (rs4494157) of *FCN3* gene were related to the variation of ficolin-3 serum concentration, they were evaluated separately and as haplotypes.

**Table 3:** Frequency of individuals with high and low ficolin-3 serum levels.

Ficolin-3 Levels	Controls N = 73	Leprosy *per se* N = 61	Lepromatous N = 45	Non lepromatous N = 16	Controls *vs*. Leprosy *per se*	Controls *vs*. Lepromatous
**High (%)**	19.2	34.4	35.5	31.2	p = 0.045^1^ OR 2.21 [95% CI 1.01–4.85]	p = 0.047^1^ OR 2.32 [95% CI 0.99–5.4]
**Low (%)**	19.2	14.7	20.0	-	n.s.	n.s.

*Statistically significant value adjusted for age, sex and ethnic group. N: number of subjects. OR: Odds ratio. CI: confidence interval. n.s: non-significant. High ficolin-3 levels was defined as >33362 ng/mL, which corresponded to the 80th percentile, and low levels as <10368 ng/mL, corresponding to the 20th percentile among controls.

The *g*.*1637del/1637C* heterozygote controls had lower ficolin-3 median concentration (3762 ng/mL) than *g*.*1637C/1637C* homozygote controls (18382 ng/mL, p = 0.023), corroborating previous published data [17,32]. Surprisingly, we did not observe the same difference between leprosy patients (p = 0.143), moreover, in both *g*.*1637del/1637C* and *g*.*1637C/1637C* genotypes, patients had higher ficolin-3 concentration than controls (S1 Table). The effect of remaining polymorphisms in ficolin-3 levels was evaluated by removing all *g*.*1637del/1637C* individuals of the analyses. In the dominant model, ficolin-3 levels were higher in leprosy *per se* and lepromatous patients with *g*.*4473A* allele when compared to *g*.*4473C/4473C* (p = 0.043 and p = 0.028, respectively). Moreover, the *g*.*3524_3532ins* and *g*.*4473A* alleles were associated with higher ficolin-3 levels in leprosy patients, compared to controls (p = 0.042, p = 0.040; respectively). Under a recessive model, homozygous genotypes for rarer alleles (*g*.*3524_3532ins* and *g*.*4473A*) seem to lead to increased ficolin-3 in patients when compared to controls. All results were adjusted for demographic factors by logistic regression (S1 Table).

The same pattern of associations was observed in the haplotypes, with ficolin-3 levels being higher in leprosy patients with the *2B1 haplotype (C*ins*A), than in those without it (32795 vs. 21958 ng/mL, p = 0.033; excluding individuals with the deletion in exon 5), and than in *2B1 controls (20790 ng/ml; p = 0.032). Similarly, lepromatous patients with the *2B1 haplotype had higher ficolin-3 levels than lepromatous patients without it (35731 vs. 22294 ng/mL, p = 0.021) and than *2B1 controls (p = 0.032). There was a trend in the same direction, between controls with and without *2B1 (p = 0.080; Fig 3B).

## Discussion

There are several evidences indicating an immunoregulatory role for the pattern recognition molecules (PRMs) of the lectin pathway in the susceptibility and clinical expression of leprosy [6,11,12,33]. This is the first study addressing *FCN3* polymorphisms and ficolin-3 levels in leprosy. In previous studies, high MBL levels were shown to increase the susceptibility to lepromatous leprosy [6,34], whereas *MBL2* haplotypes/genotypes conferring low MBL levels and deficiency in complement activation, conferred resistance against the development of lepromatous and borderline leprosy [33]. Furthermore, *FCN2* and *FCN1* haplotypes have protective effects against the susceptibility to leprosy *per se* [11,12]. Thus, components of the lectin pathway seem to be good candidates as biomarkers to be associated with the host response against *M*. *leprae*.

**Fig 4:**
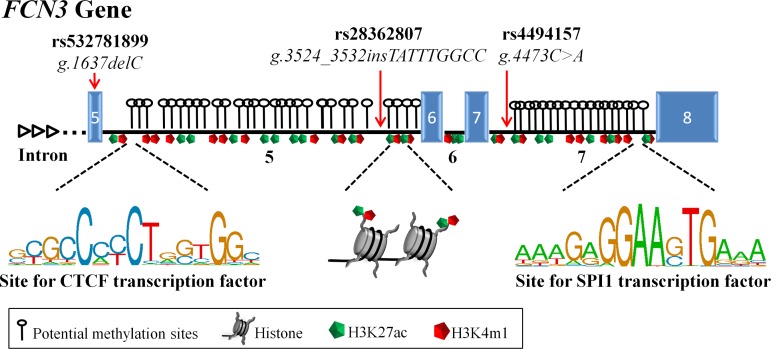
Schematic representation of the regulatory intronic region of the *FCN3* gene. Introns 5 and 7 contain many potential methylation sites (CpG islands that may act as enhancers to transcription initiation), H3K27ac and H3K4me1 histone modifications (known to flank active enhancers) and sites to transcription factors known to regulate cell activation. CTCF—CCCTC-binding factor and SPI1—Spleen focus forming virus (SFFV) Proviral Integration 1, are the most evident regulatory proteins that may bind to intron 5 and 7, respectively, data from ENCODE [37]. SNPs investigated in the present work are indicated by the red arrows. The insertion allele in intron 5 (*g*.*3524_3532insTATTTGGCC*) and the *A* at position +4473 in intron 7 (*g*.*4473A*), may increase enhancer activity in response to inflammation signals, increasing gene transcription. Exons (blue boxes) and introns are drawn to scale.

In this study, we observed that higher ficolin-3 levels were associated with the disease *per se* and with lepromatous leprosy. Higher ficolin-3 levels were also associated with a specific *FCN3* haplotype, containing an insertion in intron 5 (*g*.*3524_3532insTATTTGGCC*) and the *A* allele at position +4473 in intron 7 (*g*.*4473A*). Interesting, introns 5 and 7 contain CpG islands. They are also enriched for typical histone modifications, known to characterize active enhancers (H3K27ac—H3 acetylated at lysine 27 and H3K4me1- H3 monomethylated at lysine 4) [35,36]. Different regulatory proteins (such as CTCF—CCCTC-binding factor, SPI1—Spleen focus forming virus (SFFV) Proviral Integration 1 and EGR1—Early growth response protein 1) bind to these intronic regions, as seen by chromatin immunoprecipitation assay in different cell lines (such as NHLF—lung fibroblasts, BJ—skin fibroblast and HMC—cardiac myocytes) [37]. Variants within these sequences, as those investigated here (or others strongly linked), may increase enhancer activity in response to inflammation signals, causing enhanced gene transcription and higher protein levels (Fig 4). This would explain why we only found clear evidence for an association in patients, which probably present an inflammatory response, observing only a trend in healthy individuals. On the other hand, we cannot dismiss the possibility that binding of regulatory proteins in these sites could modulate splicing of the alternative exon 4, whose inclusion in the most abundant *FCN3* transcript leads to a longer collagenous tail.

High ficolin-3 levels have been previously reported in the sera of systemic lupus erythematosus patients [24], children with acute leukemia [21], ovarian cancer patients [25] and associated with graft loss in kidney transplant recipients [26], indicating a probable pathogenetic role for high ficolin-3 concentration in these disorders. We hypothesize that high ficolin-3 levels in leprosy patients probably play an unfavorable role by facilitating *M*. *leprae* dissemination. *M*. *leprae* may explore complement activation and opsonization induced by PRMs as one of the invasion mechanisms of macrophages and consequent evasion of the immune system [38]. Indeed, lectin pathway PRMs, including MBL (mannose-binding lectin) and ficolin-2, were shown to bind to mycobacteria leading to MASP2 (MBL-associated serine proteases) activation [38]. Although no direct binding of ficolin-3 on *M*. *bovis* BCG cell surface was found [38], it is know that the mycobacterial cell walls comprise long polymers of N-acetyl glucosamine (GlcNAc) [39], which is a ligand for ficolins [40], and could therefore be a potential target for ficolin-3.

The effect of the *g*.*1637delC* (rs532781899) polymorphism reducing ficolin-3 serum concentration [17] was only evident in controls. This is most probably a sampling effect, because among the two heterozygous *g*.*1637del/1637C* patients, one also carried the *g*.*3524_3532ins* and *g*.*4473A* alleles, associated with increased ficolin-3 concentration. Thus, whereas the *FCN3* transcript with the *g*.*1637del* allele would produce a non-functional protein in this individual, the g.*3524_3532ins* and g.*4473A* alleles, combined in a haplotype harboring the wild type allele (*g*.*1637C*), form a functional protein and lead to high ficolin-3 concentration in this patient (21337 ng/mL), elevating the ficolin-3 mean level in the heterozygous *g*.*1637del/1637C* patients.

In conclusion, we identified high concentration of ficolin-3 in leprosy patients, associated with *FCN3* polymorphisms present in introns 5 and 7. We suggest that high ficolin-3 levels increase the susceptibility to leprosy playing an unfavorable role in these patients by favoring *M*. *leprae* dissemination.

## Supporting information

S1 STROBE Checklist (DOCX)

S1 Fig Schematic representation of the amplification of *FCN3* intron 5 and 7 fragments by PCR-SSP. The use of allele specific forward (*g*.3524_3532*del or *g*.3524_3532*ins, represented in dark and light blue arrows, respectively) and reverse (*g*.4473*A or *g*.4473*C, represented in light and dark green arrows, respectively) primers allow physical haplotype phasing. Each box represents one haplotype, specified in the upper left corner. DNA sequences are showed from 5' to 3', with one strand of the genomic DNA being represented, where bold letters represent the primer annealing region and polymorphisms are shown in red. For each tested sample, the four combinations of forward and reverse primers are tested in PCR. Amplification happens only if both primers anneal perfectly to the same chromosome. If only one of the four combination result in amplification, the individual is a homozygote for that specific haplotype. If instead two combinations result in amplification, the individual is heterozygote. Amplifications are visualized in an agarose gel after electrophoresis. Another, unrelated fragment is amplified in the same PCR as a control of PCR efficiency.
(TIF)

S1 Table Ficolin-3 levels, according to *FCN3* genotypes. * Statistically significant value. ^a^Kruskal–Wallis tests. n.a. not applicable.
(DOCX)

## References

[1] Walker SL, Lockwood DNJ. Leprosy. Clin. Dermatol. 2007;25(2):165–72. 10.1016/j.clindermatol.2006.05.012 17350495

[2] World Health Organization. Weekly epidemiological record Relevé épidémiologique hebdomadaire. 2013;88:365–380.

[3] Misch EA, Berrington WR, Vary JC, Hawn TR. Leprosy and the human genome. Microbiol. Mol. Biol. Rev. 2010;74(4):589–620. 10.1128/MMBR.00025-10 21119019 PMC3008172

[4] White C, Franco-Paredes C. Leprosy in the 21st century. Clin. Microbiol. Rev. 2015;28(1):80–94. 10.1128/CMR.00079-13 25567223 PMC4284303

[5] Mira MT. Genetic host resistance and susceptibility to leprosy. Microbes Infect. 2006;8(4):1124–1131. 10.1016/j.micinf.2005.10.024 16513393

[6] Dornelles LN, Pereira-Ferrari L, Messias-Reason I. Mannan-binding lectin plasma levels in leprosy: deficiency confers protection against the lepromatous but not the tuberculoid forms. Clin. Exp. Immunol. 2006;145(3):463–8. 10.1111/j.1365-2249.2006.03161.x 16907914 PMC1809702

[7] Krarup A, Sørensen UBS, Matsushita M, Jensenius JC, Thiel S. Effect of capsulation of opportunistic pathogenic bacteria on binding of the pattern recognition molecules Mannan-binding lectin, L-ficolin, and H-ficolin. Infect. Immun. 2005;73(2):1052–1060. 10.1128/IAI.73.2.1052-1060.2005 15664949 PMC547010

[8] Sahagún-Ruiz A, Breda LCD, Castiblanco Valencia MM, Elias WP, Munthe-Fog L, Garred P, et al Studies of the binding of ficolin-2 and ficolin-3 from the complement lectin pathway to Leptospira biflexa, Pasteurella pneumotropica and Diarrheagenic Escherichia coli. Immunobiology. 2015;220(10):1177–1185. 10.1016/j.imbio.2015.06.001 26074063

[9] Mason CP, Tarr AW. Human lectins and their roles in viral infections. Molecules. 2015;20(2):2229–71. 10.3390/molecules20022229 25642836 PMC6272597

[10] Garred P, Honoré C, Ma YJ, Rørvig S, Cowland J, Borregaard N, et al The genetics of ficolins. J. Innate Immun. 2010;2(1):3–16. 10.1159/000242419 20375618

[11] De Messias‐Reason I, Kremsner PG, Kun JFJ. Functional Haplotypes That Produce Normal Ficolin‐2 Levels Protect against Clinical Leprosy. J. Infect. Dis. 2009;199(6):801–804. 19434912 10.1086/597070

[12] Boldt ABW, Sanchez MIN, Stahlke ERS, Steffensen R, Thiel S, Jensenius JC, et al Susceptibility to leprosy is associated with M-ficolin polymorphisms. J. Clin. Immunol. 2013;33(1):210–9. 10.1007/s10875-012-9770-4 22941510

[13] Genotype-Tissue Expression (GTEx), 2003–2012. Database: [Internt]. Accessed: http://www.gtexportal.org/home/gene/FCN3.

[14] Ensembl genome browser. Database: [Internet]. Accessed: http://www.ensembl.org/Homo_sapiens/Gene/Variation_Gene/Table?db=core;g=ENSG00000142748;r=1:27369112-27374824.

[15] Hummelshoj T, Fog LM, Madsen HO, Sim RB, Garred P. Comparative study of the human ficolins reveals unique features of Ficolin-3 (Hakata antigen). Mol. Immunol. 2008;45(6):1623–1632. 10.1016/j.molimm.2007.10.006 18006063

[16] Michalski M, Świerzko A St., Pągowska-Klimek I, Niemir ZI, Mazerant K, Domżalska-Popadiuk I, et al Primary Ficolin-3 deficiency–Is it associated with increased susceptibility to infections? Immunobiology. 2015;220:711–713. 10.1016/j.imbio.2015.01.003 25662573

[17] Munthe-Fog L, Hummelshøj T, Ma YJ, Hansen BE, Koch C, Madsen HO, et al Characterization of a polymorphism in the coding sequence of FCN3 resulting in a Ficolin-3 (Hakata antigen) deficiency state. Mol. Immunol. 2008;45(9):2660–2666. 10.1016/j.molimm.2007.12.012 18261799

[18] Munthe-fog L, Sc M, Hummelshøj T, Ph D, Honoré C, Madsen HO, et al. Immunodeficiency Associated with FCN3 Mutation and Ficolin-3 Deficiency. 2009;2637–2644. 10.1056/NEJMoa0900381 19535802

[19] Sallenbach S, Thiel S, Aebi C, Otth M, Bigler S, Jensenius JC, et al Serum concentrations of lectin-pathway components in healthy neonates, children and adults: mannan-binding lectin (MBL), M-, L-, and H-ficolin, and MBL-associated serine protease-2 (MASP-2). Pediatr. Allergy Immunol. 2011;22(4):424–30. 10.1111/j.1399-3038.2010.01104.x 21226765

[20] Svendsen CB, Hummelshøj T, Munthe-Fog L, Milman N, Garred P, Laursen I a., et al Ficolins and Mannose-Binding Lectin in Danish patients with sarcoidosis. Respir. Med. 2008;102(9):1237–1242. 10.1016/j.rmed.2008.04.012 18585026

[21] Schlapbach LJ, Aebi C, Hansen AG, Hirt A, Jensenius JC, Ammann RA. H-ficolin serum concentration and susceptibility to fever and neutropenia in paediatric cancer patients. Clin. Exp. Immunol. 2009;157(1):83–9. 10.1111/j.1365-2249.2009.03957.x 19659773 PMC2710595

[22] Schaffer T, Flogerzi B, Schoepfer AM, Seibold F, Müller S. Increased titers of anti-Saccharomyces cerevisiae antibodies in Crohn’s disease patients with reduced H-ficolin levels but normal MASP-2 activity. J. Crohns. Colitis. 2013;7(1):1–10. 22445443 10.1016/j.crohns.2012.02.013

[23] Prohászka Z, Munthe-Fog L, Ueland T, Gombos T, Yndestad A, Förhécz Z, et al Association of Ficolin-3 with Severity and Outcome of Chronic Heart Failure. PLoS One. 2013;8(4). 10.1371/journal.pone.0060976 PMC3626638 23596511

[24] Andersen T, Munthe-Fog L, Garred P, Jacobsen S. Serum levels of ficolin-3 (Hakata antigen) in patients with systemic lupus erythematosus. J. Rheumatol. 2009;36(4):757–759. 10.3899/jrheum.080361 19208603

[25] Szala A, Sawicki S, Swierzko AS, Szemraj J, Sniadecki M, Michalski M, et al Ficolin-2 and ficolin-3 in women with malignant and benign ovarian tumours. Cancer Immunol. Immunother. 2013;62(8):1411–9. 10.1007/s00262-013-1445-3 23744477 PMC3717161

[26] Smedbråten Y V, Sagedal S, Mjøen G, Hartmann A, Fagerland MW, Rollag H, et al High ficolin-3 level at the time of transplantation is an independent risk factor for graft loss in kidney transplant recipients. Transplantation. 2015;99(4):791–6. 10.1097/TP.0000000000000422 25222012

[27] Ridley DS, Jopling WH. Classification of leprosy according to immunity. A five-group system. Int. J. Lepr. Other Mycobact. Dis. 1966;34(3):255–73. 5950347

[28] Boldt ABW, Petzl-Erler ML. A new strategy for mannose-binding lectin gene haplotyping. Hum. Mutat. 2002;19(3):296–306. 10.1002/humu.10051 11857747

[29] National Center for Biotechnology Information (NCBI). Database: [Internet]. Accessed: www.ncbi.nlm.nih.gov.

[30] Nebert DW. Proposal for an Allele nomenclature system based on the evolutionary divergence of haplotypes. Hum. Mutat. 2002;20(6):463–472. 10.1002/humu.10143 12442271

[31] Boldt ABW, Messias-Reason IJ, Meyer D, Schrago CG, Lang F, Lell B, et al Phylogenetic nomenclature and evolution of mannose-binding lectin (MBL2) haplotypes. BMC Genet. 2010;11:38 10.1186/1471-2156-11-38 20465856 PMC2885306

[32] Michalski M, Szala A, St. Swierzko A, Lukasiewicz J, Maciejewska A, Kilpatrick DC, et al H-ficolin (ficolin-3) concentrations and FCN3 gene polymorphism in neonates. Immunobiology. 2012;217(7):730–737. 10.1016/j.imbio.2011.12.004 22226667

[33] De Messias-Reason IJ, Boldt ABW, Moraes Braga AC, Von Rosen Seeling Stahlke E, Dornelles L, Pereira-Ferrari L, et al The association between mannan-binding lectin gene polymorphism and clinical leprosy: new insight into an old paradigm. J. Infect. Dis. 2007;196(9):1379–1385. 10.1086/521627 17922403

[34] Garred P, Harboe M, Oettinger T, Koch C, Svejgaard A. Dual role of mannan-binding protein in infections: another case of heterosis? Eur. J. Immunogenet. 1994;21(2):125–31. 9098426 10.1111/j.1744-313x.1994.tb00183.x

[35] Deaton A, Bird A. CpG islands and the regulation of transcription. Genes Dev. 2011;25(10):1010–1022. 10.1101/gad.2037511 21576262 PMC3093116

[36] Shlyueva D, Stampfel G, Stark A. Transcriptional enhancers: from properties to genome-wide predictions. Nat. Rev. Genet. 2014;15(4):272–86. 10.1038/nrg3682 24614317

[37] Encyclopedia of DNA Elements (ENCODE). Database [Internet]. Accessed: http://genome.ucsc.edu/cgi-bin/hgTracks?db=hg19&lastVirtModeType=default&lastVirtModeExtraState=&virtModeType=default&virtMode=0&nonVirtPosition=&position=chr1%3A27697287-27699264&hgsid=502309425_rYKsJ2LDapXZnKsbIuMaheJfT09r.

[38] Carroll M V., Lack N, Sim E, Krarup A, Sim RB. Multiple routes of complement activation by Mycobacterium bovis BCG. Mol. Immunol. 2009;46(16):3367–3378. 10.1016/j.molimm.2009.07.015 19698993

[39] Kieser KJ, Rubin EJ. How sisters grow apart: mycobacterial growth and division. Nat. Rev. Microbiol. 2014;12(8):550–562. 10.1038/nrmicro3299 24998739 PMC6556109

[40] Matsushita M. Ficolins: Complement-activating lectins involved in innate immunity. J. Innate Immun. 2009;2(1):24–32. 10.1159/000228160 20375620

