# Supplementary figures and images for: Association of a new FCN3 haplotype with high ficolin-3 levels in leprosy
Source: PLoS Negl Trop Dis. 2017 Feb 27;11(2):e0005409. doi: 10.1371/journal.pntd.0005409 (PMC5344521; doi:10.1371/journal.pntd.0005409)

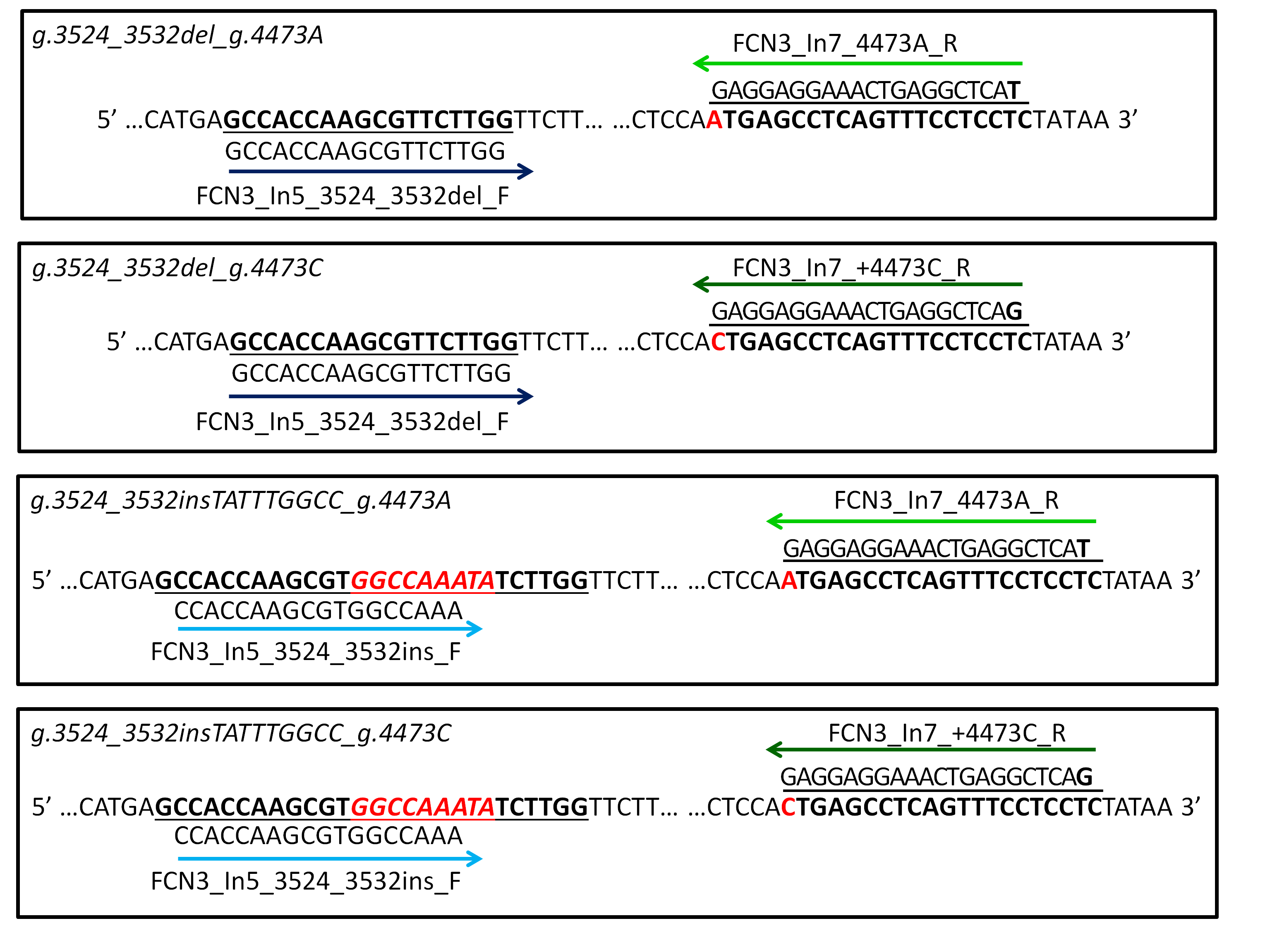

Supplement: S1 Fig — The use of allele specific forward (g.3524_3532*del or g.3524_3532*ins, represented in dark and light blue arrows, respectively) and reverse (g.4473*A or g.4473*C, represented in light and dark green arrows, respectively) primers allow physical haplotype phasing. Each box represents one haplotype, specified in the upper left corner. DNA sequences are showed from 5' to 3', with one strand of the genomic DNA being represented, where bold letters represent the primer annealing region and polymorphisms are shown in red. For each tested sample, the four combinations of forward and reverse primers are tested in PCR. Amplification happens only if both primers anneal perfectly to the same chromosome. If only one of the four combination result in amplification, the individual is a homozygote for that specific haplotype. If instead two combinations result in amplification, the individual is heterozygote. Amplifications are visualized in an agarose gel after electrophoresis. Another, unrelated fragment is amplified in the same PCR as a control of PCR efficiency. (TIF) [file pntd.0005409.s002.tif]
