# Supplementary material for: Association of a new FCN3 haplotype with high ficolin-3 levels in leprosy
Source: PLoS Negl Trop Dis. 2017 Feb 27;11(2):e0005409. doi: 10.1371/journal.pntd.0005409 (PMC5344521; doi:10.1371/journal.pntd.0005409)
Supplement: S1 Table — * Statistically significant value. aKruskal–Wallis tests. n.a. not applicable. (DOCX) [file pntd.0005409.s003.docx]

**S1 Table. Ficolin-3 levels, according to *FCN3* genotypes.**

| **Genotype** | **Controls** | **Leprosy *per se*** | **Controls x**  **Leprosy *per se*** | **Lepromatous** | **Non lepromatous** | **Lepromatous x**  **Non lepromatous** |
| --- | --- | --- | --- | --- | --- | --- |
| **Dominant Model** | Median ng/mL [range] | Median ng/mL [range] |  | Median ng/mL [range] | Median ng/mL [range] |  |
| **rs532781899** |  |  |  |  |  |  |
| *g.1637del/1637C* | 3762 [3369-4156] | 13726 [6117-21337] | p<0.001* | 6117 | 21337 | p=0.417^1^ |
| *g.1637C/1637C* | 18382 [3129-60300] | 26164 [3682-60300] | p=0.02* | 28295 [3682-60300] | 21958 [11510-56473] |  |
|  | p=0.023* | p=0.143 |  | n.a. | n.a. |  |
| **rs28362807** |  |  |  |  |  |  |
| *g.3524_3532ins/_* | 19941 [9708-60300] | 32795 [9422-58995] | p=0.042*; OR=42.31 | 33687 [9422-58995] | 23903 [18467-43602] | p=0.239 |
| *g.3524_3532del/3524_3532del* | 17122 [4663-48609] | 21958 [3769-60300] | p=0.312 | 22857 [3769-60300] | 20381 [15075-56473] | p=0.393 |
|  | p=0.166 | p=0.06 |  | p=0.056 | p=0.51 |  |
| **rs4494157** |  |  |  |  |  |  |
| *g.4473A/_* | 20790 [9708-60300] | 32795 [9422-58995] | p=0.032*; OR=51.14 | 35731 [9422-58995] | 23903 [21337-26034] | p=0.135 |
| *g.4473C/4473C* | 16815 [3129-48609] | 21958 [3769-60300] | p=0.256 | 22294 [3769-60300] | 21169 [15075-56473] | p=0.64 |
|  | p=0.180 | p=0.043*; OR=11.13 |  | p=0.028*; OR=15.8 | p=0.798 |  |
| **Recessive Model** |  |  |  |  |  |  |
| **rs28362807** |  |  |  |  |  |  |
| *g.3524_3532ins/3524_3532ins* | 28391 [21825-34957] | 40321 [33687-42993] | p=0.028*^a^ | 40321[33687-42993] | - | p=0.349^a^ |
| *g.3524_3532del/_* | 18248 [3129-60300] | 25738 [3682-60300] |  | 26016[3682-60300] | 21865 [15075-56473] |  |
|  | n.a. | p=0.152 |  | p=0.166 | n.a. |  |
| **rs4494157** |  |  |  |  |  |  |
| *g.4473A/4473A* | 28391[21825-34957] | 40321 [33687-42993] | p=0.028*^a^ | 40321[33687-42993] | - | p=0.349^a^ |
| *g.4473C/_* | 18248 [3129-60300] | 25738 [3682-60300] |  | 26016[3682-60300] | 21865 [15075-56473] |  |
|  | n.a. | p=0.152 |  | p=0.166 | n.a. |  |
